# Antiviral Potential of Selected Medicinal Herbs and Their Isolated Natural Products

**DOI:** 10.1155/2021/7872406

**Published:** 2021-12-08

**Authors:** W. P. R. T. Perera, Janitha A. Liyanage, K. G. C. Dissanayake, Hiruni Gunathilaka, W. M. T. D. N. Weerakoon, D. N. Wanigasekara, W. S. K. Fernando, R. M. H. Rajapaksha, R. P. Liyanage, Bingun T. Perera

**Affiliations:** ^1^Department of Indigenous Medical Resources, Faculty of Indigenous Health Sciences and Technology, Gampaha Wickramarachchi University of Indigenous Medicine, Sri Lanka; ^2^Department of Chemistry, Faculty of Science, University of Kelaniya, Sri Lanka; ^3^Department of Cikitsa, Faculty of Indigenous Medicine, Gampaha Wickramarachchi University of Indigenous Medicine, Sri Lanka; ^4^Department of Rogavignana, Faculty of Indigenous Medicine, Gampaha Wickramarachchi University of Indigenous Medicine, Sri Lanka; ^5^Department of Biochemistry, Faculty of Medicine, University of Ruhuna, Sri Lanka; ^6^Department of Chemistry, University of Iowa, USA

## Abstract

Viruses are responsible for a variety of human pathogenesis. Owing to the enhancement of the world population, global travel, and rapid urbanization, and infectious outbreaks, a critical threat has been generated to public health, as preventive vaccines and antiviral therapy are not available. Herbal medicines and refined natural products have resources for the development of novel antiviral drugs. These natural agents have shed light on preventive vaccine development and antiviral therapies. This review intends to discuss the antiviral activities of plant extracts and some isolated plant natural products based on mainly preclinical (in vitro and in vivo) studies. Twenty medicinal herbs were selected for the discussion, and those are commonly recognized antiviral medicinal plants in Ayurveda (*Zingiber officinale*, *Caesalpinia bonducella*, *Allium sativum*, *Glycyrrhiza glabra*, *Ferula assafoetida*, *Gymnema sylvestre*, *Gossypium herbaceum*, *Phyllanthus niruri*, *Trachyspermum ammi*, *Withania somnifera*, *Andrographis paniculata*, *Centella asiatica*, *Curcuma longa*, *Woodfordia fruticose*, *Phyllanthus emblica*, *Terminalia chebula*, *Tamarindus indica*, *Terminalia arjuna*, *Azadirachta indica*, and *Ficus religiosa)*. However, many viruses remain without successful immunization and only a few antiviral drugs have been approved for clinical use. Hence, the development of novel antiviral drugs is much significant and natural products are excellent sources for such drug developments. In this review, we summarize the antiviral actions of selected plant extracts and some isolated natural products of the medicinal herbs.

## 1. Introduction

Many herbal remedies individually or in combination, as well as various formulations such as leaf powder, pastes, decoctions, infusions, and pills, have been recommended for different medical treatments, and numerous biologically active agents have been recognized for their various therapeutic functions. In the last few decades, thorough studies of phytochemicals for antiviral activities have assumed huge significance. A wide range of active phytochemicals, including flavonoids, terpenoids, organosulphur compounds, limonoids, lignans, sulfides, polyphenols, coumarins, saponins, chlorophyllins, furyl compounds, alkaloids, polyins, thiophenes, proteins, and peptides, has shown to have therapeutic applications against various genetically and functionally diverse viruses [1–5].

Medicinal plants have been used in the world, but their widespread use has been limited to the Asian region of the world such as China, India, Japan, Sri Lanka, and Thailand as well as some other African nations. However, developed countries are also working towards encouraging the use of natural medicinal products based on plant products in their healthcare systems [6].

Molecular pathways related to the antiviral effects of plant extracts may vary from virus to virus. However, the ability of plant extract to improve the inherent antiviral protection of the human body requires an intricate immune system [7, 8].

Plant-derived preparations have been made for centuries used to combat many diseases. Over the past few decades, natural phytochemicals have been tested for antiviral properties more specifically. However, owing to viral infections is becoming a significant threat to humans, further research work is still needed to gain effective knowledge about viral infections. In addition, medicinal plants are increasingly being suggested as suitable alternative sources of antiviral agents. Developing suitable in vitro pharmacodynamics screening techniques may contribute to the rapid identification of potential bioactive plants. Unfortunately, many antiviral compounds currently in clinical use have a narrow range of activity, minimal therapeutic utility, and variable toxicity [9–13].

Enhancement of the viral resistivity on the existing antiviral drugs and therapies is another challenge that the world population will face in the near future. In addition, the need to develop new antiviral drugs from the bioactive compounds of the plant is further exacerbated by the fact that viral infections are now recognized as the second most significant known cause of human cancer [14]. The main viral controlling mechanisms of bioactive compounds in medicinal herbs may include antioxidant activity, scavenging ability, inhibiting DNA, RNA synthesis, inhibiting viral entry, or inhibiting viral reproduction [15]. A significant number of plant-derived molecules exhibit antiviral activity, but their mechanical action needs to be explored. As medicinal plants have an infinite range of chemical constituents, they could be used to inhibit the replication of both DNA and RNA viruses [16].

Our intention of this review is to summarize the literature on antiviral bioactive compounds, discovered antiviral mechanisms, and antiviral actions of aqueous extracts of commonly used antiviral herbs in Ayurveda medicine.

## 2. Methodology

Literature searches were done regarding antiviral actions of the medicinal herbs in PubMed, PMC, and ScienceDirect. Furthermore, antiviral bioactive compounds of the selected herbs and their mechanisms were searched in some popular search engines like Google, Google Scholar, to gather secondary data. All materials, regardless of sources, were reviewed, and the review framework was developed to represent the information available. Chemical structures were prepared by using ChemDraw software.

## 3. Results and Discussion

### 3.1. Zingiber officinale

Ginger (*Zingiber officinale*), a member of the *Zingiberaceae* family and the genus *Zingiber*, has long been widely consumed as a spice and herbal medicine [17].

**Figure 1:**
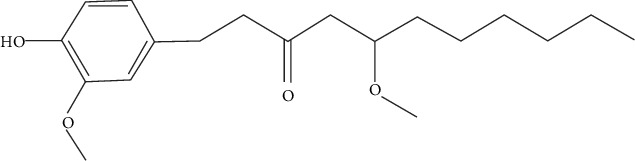
Chemical structure of the gingerols, a major phenolic compound that identified as the dominant constituent of both fresh and dried gingers.

Gingerols (Figure 1) and shogaols are the phenolic compounds that are mainly responsible for the health benefits of dinger. Related research has shown that ginger has numerous biological activities, including antioxidant, anti-inflammatory, antimicrobial, anticancer, neuroprotective, cardiovascular, gastrointestinal, antiobesity, antidiabetic, and antinausea activities [18].

Fresh ginger was found to inhibit plaque formation induced by the human respiratory syncytial virus (HSRV) in respiratory tract cell lineages, and it is successful in blocking internalization and viral attachment. This analysis clearly showed that fresh ginger had antiviral activity on both HEp2 and A549 cells against HRSV. 300 milligrams/mL in both cell types, fresh ginger reduced HRSV infection by more than 70%. In addition to dried ginger, cells protect around 20% from viral plaque formation in only HEp-2 cells [19]. It reveals that the phytochemistry of ginger plays a major role in suppressing viral activities; once it is dried, antiviral activities are reduced due to lacking secondary metabolites.

The fresh ginger comprises [6]-, [8]-, and [10]-gingerols as the major pungent, and [4]- and [5]-gingerols contain trace amounts [20]. However, [6]-, [8]-, and [10]-gingerols are turned into shogaols during the commercial drying process [21], and dried ginger has not shown antiviral capabilities in most of the studies. Hence, it can be concluded that gingerols would be the most significant phytocompound inhibiting HRSV activities. But activity or the mechanism of the gingerols against HRSV has not been proven experimentally. In addition, HRSV binds and penetrates the G proteins and F- fusion proteins, respectively, into the cells. Hence, fresh ginger should have a similar effect on HEp-2 and A549 cells [22].

### 3.2. Caesalpinia bonducella

*Caesalpinia bonducella* is often remembered as a “fever nut.” The nut and nicker nut of Bondoc belong to the family of *Caesalpiniaceae* and have been recorded in Folklore Medicine and the ancient scriptures of Ayurveda. The seed of the *C. bonducella* contains Bonducin as the major active molecule. Apart from that, saponins and terpenoids are also known as other major secondary metabolites found in seeds. The shell contains fatty oil, starch, sucrose, phytosterols, stearic, palmitic, oleic, linoleic, linolenic, and a mixture of unsaturated fatty acids of low molecular weights [23, 24].

**Figure 2:**
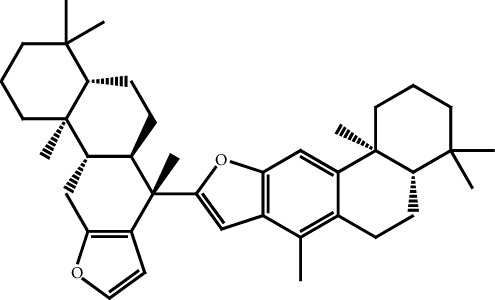
Chemical structure of the Taepeenin J, cassane-type diterpenes readily found in seeds of Caesalpinia species.

*C. bonducella* root extract has shown antiviral activity against the vaccinia virus. But details about viral controlling mechanisms and related phytocompounds have not been pointed out [25]. Further, phytochemicals in the seeds *C. bonducella* which are responsible for the antiviral activities and have not been identified separately and viral controlling mechanisms also have not clearly been reported in the literature. However, one of the computational investigations has discovered Taepeenin J, as a promising candidate for the receptor inhibition process to mitigate cytokine storms due to the infection of the SARS-COV-19 virus. Taepeenin j (Figure 2) is 1 of 12 cassane-type diterpenes readily found in seeds of *Caesalpinia* species which belongs to the family of *Caesalpiniaceae* [26]. Hence, it can be suggested that more attention is needed to pay for the antiviral activities of the seeds related to the family of *Caesalpiniaceae*.

### 3.3. Allium sativum

*Allium sativum*, also known as garlic, one of the most common herbal remedies used in human history, dates back to ancient cultures [27]. The main bioactive compound of the *A. sativum* is allicin, and garlic extract with the allicin has been shown antiviral activities in vitro and in vivo due to sulfur-containing compounds such as allicin, diallyl disulfide, and diallyl trisulfide that react with thiol groups of various enzymes which are critical for microorganism surveillance [28, 29].

There are so many preclinical investigations that have been done to study antiviral activities of the different aqueous extracts of the *A. sativum*. According to one of the toxicity assays that were carried out using chicken embryos, an aqueous extract of the *A. sativum* has performed an antiviral effect against coronavirus species [30].

Meanwhile, another plaque reduction study suggests that aqueous extract in gold nanoparticles of *A. sativum* showed antiviral activity against the measles virus (MeV) by direct inhibition of the virus via blocking the virus entry [31]. Herpes simplex virus- (HSV-) 1 and 2 viral inhibition assays also have been performed by direct preinfection incubation and plaque reduction assays with Vero and HeLa cell propagation methods. Those performances are also shown by aqueous extract in gold nanoparticles of *A. sativum*, and the proposed mechanism is inhibition of virus entry via disruption of the viral envelope and cell membrane [32]. Apart from that, early antigen assay has been done with extract of *A. sativum* against HCMV, human cytomegalovirus, and identified controlling mechanisms have been explained as inhibition of viral replication and boosting the immune response [33]. In addition to the fresh extract of garlic, the oil form of garlic also plays an important role in the inhibition of viruses. An antiviral experiment has been done using garlic oil against IBV influenza A virus-H1N1 via assessment of cytopathic effects in IAV-H1N1-infected cells and discovered reduced, visible cytopathic effects in IAV-H1N1-infected cells [34]. Apart from water extract and oil extracts of the *A. sativum*, the n-hexane extract also has examined for HIV reverse transcriptase inhibitory assay and positive results were obtained [35].

The organosulfur compounds extracted from *A. sativum* also have been investigated for different antiviral experiments, and its significant inhibition actions have been performed on different disease-forming viral species in human (Table 1).

### 3.4. Glycyrrhiza glabra

*Glycyrrhiza glabra* (licorice) has been well known in pharmacy for a long time. In the old ones, it was considered a first-class drug in Chinese pharmacy and the rejuvenating quality was attributed to it when ingested for long periods. Licorice has been used in ancient Egypt, Greece, and Rome [40]. *Glycyrrhiza glabra* contains different secondary metabolites that can be extracted from its roots and stem, and those are hydrophilic as well as biologically active. Glycyrrhizin is a major triterpenoid present in *G. glabra* which is responsible for the sweetness and taste of its roots [41].

**Figure 3:**
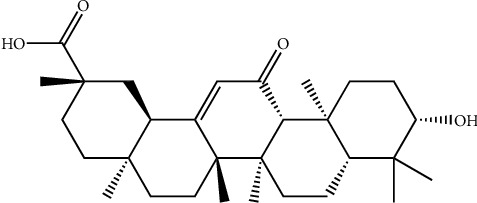
18*β*-glycyrrhetinic acid (18*β*-GA), the active phytochemicals in licorice which performed antiviral activity against HRSV.

Meanwhile, one of the significant antiviral studies reported that hot water extract preparations of the licorice have performed antiviral efficacy against the human respiratory syncytial virus (HRSV). Glycyrrhizin and 18*β*-glycyrrhetinic acid (18*β*-GA), the active phytochemicals in licorice, have been examined by plaque reduction assay in both human upper (HEp-2) and low (A549) respiratory tract cell lines. According to the results, one of the preparations of licorice called Radix Glycyrrhizae inhibited the HRSV induced plaque formation in both HEp-2 and A549 cell lines and those actions may be due to its inhibition of viral attachment and penetration into the host cells. Apart from that, 18*β*-GA (Figure 3) also showed a potent anti-HRSV activity [12, 41, 42].

Other than HRSV, secondary metabolites of the *G. glabra* showed effective antiviral actions against influenza virus A2 (H2N2). According to studies conducted by Utsunomiya and his colleagues in 1997, it is suggested that the antiviral efficacy of glycyrrhizin is essentially due to the stimulation of *β*-interferon released by T-cells. In this analysis, mice were selected to be infected with the influenza A2 (H2N2) virus. They were unable to tolerate and survive 10 times the lethal dose (LD50) of the virus. When treated with 10 mg glycyrrhizin/kg at different periods, such as on the day before, on the day after, and on the fourth day after infection, complete survival was observed [43].

**Figure 4:**
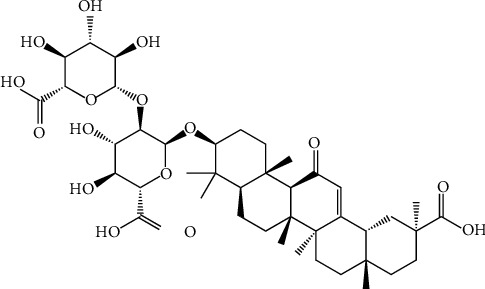
Chemical structure of the glycyrrhizin, natural saponin composed in *G. glabra* root/stem extract.

Glycyrrhizin (Figure 4) which is extracted from licorice as triterpenoid has performed more significant antiviral activates on some DNA and RNA-based viruses. It was able to inhibit the plaque formation of the herpes simplex virus (HSV-1 and HSV-2), and some inhibitory activity showed on polio type 1, 2, and 3 as well as measles viral species. Further, glycyrrhizin is more effective in the regulation of viral replication and can be used as a prophylactic measure. It was also used to prevent the replication of the severe acute respiratory syndrome (SARS) coronavirus [44].

### 3.5. Ferula assafoetida

The oligo gum resins of *Ferula assafoetida* contain mainly five forms of sesquiterpene cumarine, namely, conferone, badrakemin, feslol, isosamarcandin, and samarcandin. These compounds vary depending on the presence or absence of the hydroxyl group, the position of the double bond, and the configuration of the chiral center. This means the main distinction between these compounds is due to the different sesquiterpene units [45, 46].

**Figure 5:**
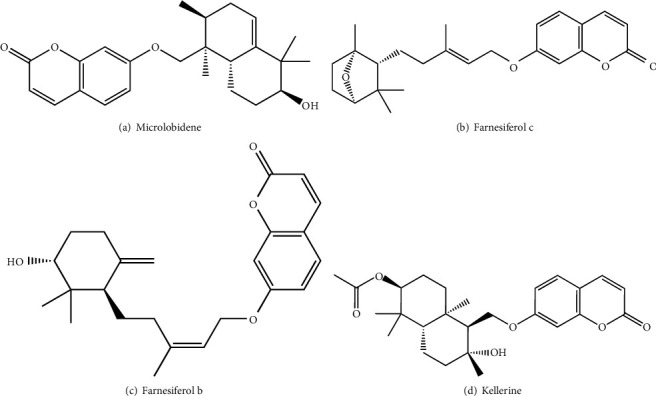
Sesquiterpene cumarines isolated from oligo-gum resin of *Ferula assafoetida* showed antiviral properties against rhinovirus (HSV).

According to one study from the antiviral study group, CPE (cytopathic effect) inhibitory studies were done with *Ferula assafoetida* gum resin and its isolated sesquiterpene: Microlobidene (1), Farnesiferol C (2), Farnesiferol B (3), and Kellerin (4) (Figure 5). They were performed in HeLa cells with pleconaril-sensitive HRV-1A, 2, 14, and 16 in HeLa cells. Findings from CPE-inhibitory assays showed it has a dose-dependent antiviral activity against HRV-2 for asafetida gum resin. However, prevention of rhinovirus adsorption (HRV-2) is the mode of inhibition action [47]. Farnesiferol C and Farnesiferol B can be identified as novel chemical scaffolds having HRV-2 inhibiting potential where the micromolar range is low, while asafetida gum resin has been used for disorders of the upper respiratory tract [48].

**Figure 6:**
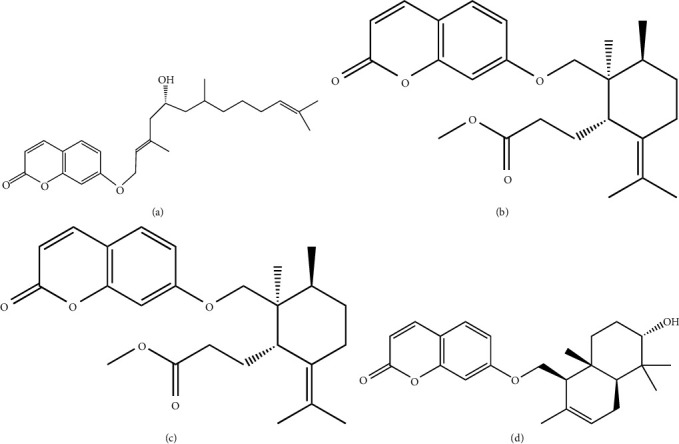
Sesquiterpene cumarines isolated from oligo-gum resin of *Ferula assafoetida* against showed antiviral properties against influenza A H1N1 virus: (a) 8′-acetoxy-5′S-hydroxyumbelliprenin, (b) methyl galbanate, (c) galbanic acid, and (d) conferol.

Sesquiterpene coumarins, which are isolated from the resin of the *F. assafoetida*, have been demonstrated antiviral activity against the influenza A virus (H1N1). According to the IC_50_ value calculations of this experiment, 8′-acetoxy-5′S-hydroxyumbelliprenin, methyl galbanate, galbanic acid, Farnesiferol C, Farnesiferol A, and conferol (Figure 6) showed greater potency against influenza A virus (H1N1) (IC_50_ 0.26-0.86 *μ*g/mL) than amantadine (IC_50_ 0.92 *μ*g/mL), (positive control) [49],

### 3.6. Gymnema sylvestre

*Gymnema sylvestre* is native to central and western India, tropical Africa, and Australia [50]. The most abundantly found phytochemical groups in *G. sylvestre* leaves are triterpene and saponins. And other plant constituents are flavones, anthraquinones, hentriacontane, pentatriacontane, *α* and *β*-chlorophylls, resins, d-quercitol, tartaric acid, phytin, butyric acid, formic acid, lupeol, *β*-amyrin-related glycosides, and stigmasterol. It is also positive for alkaloids, according to phytochemical investigations. The leaves of this species contain acid glycosides, anthraquinones, and their derivatives [51].

Some research studies have detected ethanolic and methanolic leaf extract activity of G. sylvestre against HIV-1, reverse transcriptase (RT) enzyme, HBV DNA polymerase enzyme, and HBsAg assays. The effect was evaluated using a nonradioactive HIV-RT colorimetric kit. The binding activity of HBsAg was evaluated using the ELISA kit and the activity of HBV DNA polymerase using the Radiometric Kit. Inhibition of 50 percent (IC50) was considered a positive activity. The methanolic extract was found to possess potent in vitro HBsAg binding, inhibition of HBV DNA polymerase, and inhibition of HIV-1 RT activity, whereas ethanolic extracts are positive for inhibition of HIV-1 RT [52].

Reports on *G. sylvestre* indicate that the plant leaves have nutritional and medicinal value in the extracts of leaf, stem, root, and fruit that are important from the research point of view [53]. And also, they have shown the antiviral properties of phytochemicals like triterpenoid saponins [54] and gymnemic acids [55].

### 3.7. Gossypium herbaceum

*Gossypium herbaceum* is known as a cotton plant, belongs to the *Malvaceae* family, and is widely distributed throughout western India, middle east countries, Africa, Central Asia, Iran, Russia, Afghanistan, and Turkey. The qualitative phytochemical investigations of these plant extracts indicate the presence of carbohydrates, saponins, glycosides, steroids, phenolic compounds such as flavonoids, and tannins [56].

**Figure 7:**
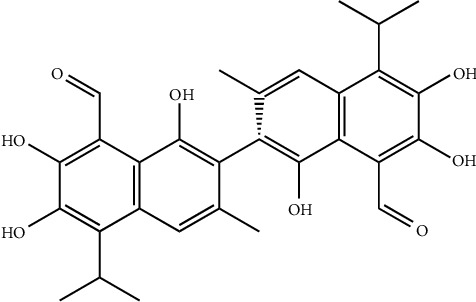
The structure of gossypol, an active antiviral compound found in the principle pigment of cotton (*Gossypium herbaceum*).

The key pigment of cotton seed was gossypol (Figure 7), a phenolic compound that was first isolated in 1899. Researchers have found that this compound has antiviral properties against enveloped viruses, including HIV-1, HSV-2, influenza, and parainfluenza [57].

Incubation of HTLV-III B strain of human immunodeficiency virus with gossypol has shown that it can prevent the recovery of viable viruses when subsequently incubate with H9-T cells [58]. Variety analogs of gossypol displayed more activity against type 1 (HIV-l) immunodeficiency virus, while the racemic mixture and both gossypol enantiomers prevented the replication of type 1 (HIV-l) human immunodeficiency virus [59].

Water extracts of *Gossypium hirsutum* leaves have also been investigated for the antiviral effect against the yellow fever virus in the tissue cell culture using Vero cells. The extracts demonstrated antiviral activity against the yellow fever virus by inhibiting the yellow fever virus in MICs of 0.079 mg/mL [60].

### 3.8. Phyllanthus niruri

*Phyllanthus niruri* is a perennial tropical traditional shrub of the *Phyllanthaceae* family with long-standing Ayurveda, Chinese, and Malay ethnomedical records. Preliminary studies from cell and animal models have provided valuable scientific evidence for its use which has been used for a wide range of diseases in South and Southeast Asian traditional medicine [61].

Antiviral activity is the most prominent among the potential therapeutic effects of *P. niruri*. Studies conducted by obtaining chronic hepatitis B patients and woodchuck hepatitis- (WHV-) infected woodchucks, which were treated with extracts of this plant, showed decreased viral antigen levels [62]. Aqueous extracts of *P. niruri* have been shown to possess significant antiviral potential and found to be a promising approach for hepatitis B carriers [63].

**Figure 8:**
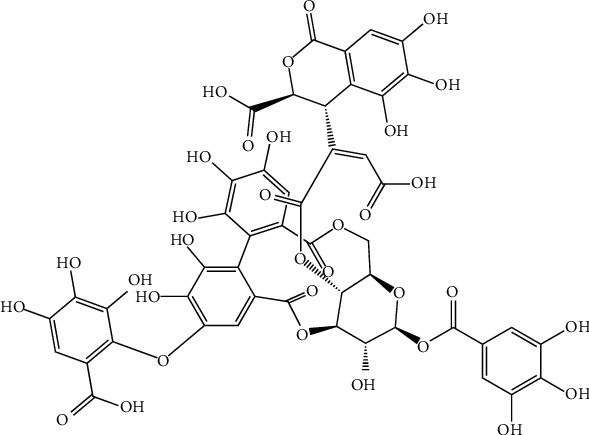
Chemical structure of the repandusinic acid, a hydrolyzable tannin, contains a significant inhibitory effect on HIV-1 reverse transcriptase.

The antiviral activity of *P. niruri* is not just limited to the hepatitis B virus, but also aqueous extracts of *P. niruri* containing repandusinic acid (Figure 8), a hydrolyzable tannin, have been shown to apply a significant inhibitory effect on HIV-1 reverse transcriptase [64]. According to the kinetic analysis, it has been suggested that repandusinic acid competitively inhibits the template primer during the process of reverse transcription [62].

In addition, various members of the *Phyllanthus* family have exhibited inhibitor activity against a wide range of viruses, such as hepatitis B virus (HBV), hepatitis C virus (HCV), human immunodeficiency virus (HIV), and herpes simplex virus (HSV) [65].

### 3.9. Trachyspermum ammi

*Trachyspermum ammi*, also known as ajwain from the *Apiaceae* family, is an old herb with various medicinal properties. The oil extracted from the seeds of *T. ammi* from the *Apiaceae* family showed remarkable antibacterial, antiviral, antifungal, antitussive, anti-inflammatory, and analgesic effects [66].

Japanese encephalitis virus (JEV) titer has been determined by plaque assay in vitro, and ajwain oil antiviral activity was quantified by a plate-reduction neutralization test (PRNT). Their data suggested that ajwain oil has possible in vitro antiviral activity against JEV. Furthermore, the active biomolecule (thymol) present in ajwain oil must be investigated in order to explore its potential for antiviral drugs in the future [67].

### 3.10. Withania somnifera

*Withania somnifera*, is a very valuable medicinal herb and commonly known as “Ashwaghanda.” It belongs to the genus of *Withania* and the family of *Solanaceae* and is found in Asian countries, including India [68]. Laboratory research has shown that there are more than 35 chemical components at the root of *W. somnifera* [69].

Biologically active chemical constituents of this plant are alkaloids (isopelletierine, anferine), steroidal lactones (withanolides, withaferins), saponins containing additional classes of acyls, and withanoloids containing glucose [70].

Many researchers studied the antiviral function of these plant extracts against the replication of the Infectious Bursal Disease virus. *W. somnifera* roots hydroalcoholic extract demonstrated inhibition of the virus at a maximum of 99.9% at its maximum nontoxic concentration, 25 *μ*g/mL in the cytopathic effect reduction assay [71].

*W. somnifera* extract also demonstrated antiviral activity against the herpes simplex type-1 virus. *W. somnifera* and *Aloe ferox* are widely used to treat sexually transmitted infections (STIs). Aqueous extracts of these two species, together with aloin which is isolated from *A. ferox*, have been tested for in vitro antiviral potential against herpes simplex virus type 1 (HSV-1). Aqueous extracts of *W. somnifera* have shown significant activity against the virus in the Vero cells of African green monkey cell culture [72].

**Figure 9:**
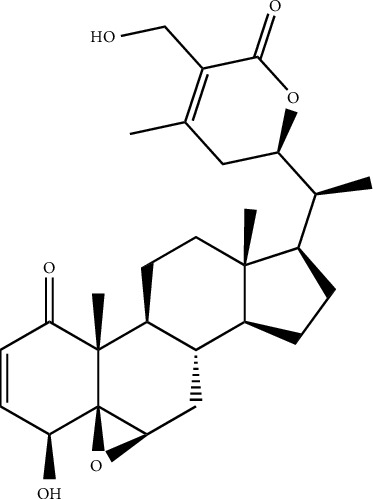
Structures of Withaferin A, a steroidal compound present in *Withania somnifera.*

The inhibitory potential of Withaferin A (Figure 9) which is a steroid present in *W. somnifera* against herpes simplex virus has also been evaluated. Researchers conducted docking and further molecular dynamic simulation studies to elucidate the mechanism of binding of prospective herbal drug withaferin A to the structure of the herpes simplex virus's DNA polymerases [73]. And also *W. somnifera* has demonstrated antiretroviral actions against HIV infections [74].

### 3.11. Andrographis Paniculata

*Andrographis paniculata* which is also known as “king of bitters” belongs to the family *Acanthaceae.* Most countries like India, China, Malaysia, and Thailand have been used this herbaceous plant to treat many illnesses from ancient times [75].

*A.s paniculata* is well known for its antiviral, antibacterial, and antifungal properties. Methanolic extracts while the aqueous extract of *A. paniculata* has tested in the H9 cell line against HIV and it had reported inhibitory activity against HIV [75].

The main constituents of *A. paniculata* are flavonoids, diterpenoids, and polyphenols. Andrographolides are the major bioactive phytoconstituent, found in *A. paniculata*.

**Figure 10:**
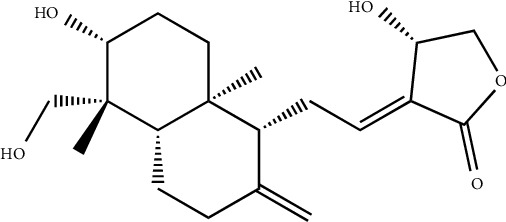
Structure of andrographolide, major phytoconstituent in *Andrographis paniculata.*

This phytoconstituent was tested for antiviral activity against herpes simplex virus (HSV) [76], human immunodeficiency virus (HIV) [77], flaviviruses [78], and pestiviruses [79]. It has been demonstrated that the ethanolic extract of *A. paniculata* (25 *μ*g/mL) and andrographolide (Figure 10) (5 *μ*g/mL) has a pronounced effect in inactivation or the inhibition of Epstein Virus (EBV) lytic proteins, Rta, Zta, and EA-D expression in P3HR1 cells.

According to a recent study, *A. paniculata* exhibits an antiviral inhibitory effect against DENV1-infected Vero E6 cells [80]. Apart from antiviral properties, it exhibits many useful properties like hypolipidemic effect, antihyperglycemic activities, antipyretic and analgesic effects, and antimalarial effects [75].

### 3.12. Centella asiatica

*Centella asiatica* is commonly known as “Gotu kola” in Sri Lanka and China, *Mandukparni* or Indian pennywort, or *jalbrahmi* in India. This herbaceous plant is also used by people in the Indonesian islands and Java as a medicinal plant [81].

The major active components of *C. asiatica* are triterpenoids (saponins) which include asiaticosides [82]. This compound is responsible for the vascular effects and wound healing effects by inhibiting the production of collagen at the wound site. Apart from that, total plant extract contains flavonoids and plant sterols [83]. Major triterpenoids include asiatic acid, asiaticoside, madecassic acid, madasiatic acid, madecassoside, and isothankunic acid [84].

Crude water extract of *C. asiatica* exhibits antiherpes simplex activity. *C. asiatica* has shown anti-HSV-1 and 2 inhibition abilities via plate inhibition assay (with median effective dose (ED_50_) which was 362 *μ*g/ml and 298 *μ*g/ml for HSV-1 and HSV-2, respectively) [85]. In addition to that, another research group has revealed methanolic and aqueous extracts of the *C. asiatica* as an excellent virucidal, prophylactic, and marked antiviral attachment activities with relevant assays against pseudorabies virus (PrV) as well as it was discovered that methanolic extract of *C. asiatica* was acting as most active antiviral attachment agents with percent cell viability up to 60% among other tested plant extracts [86].

Though there are fewer reports regarding the antiviral properties of *C. asiatica*, there are many publications regarding other pharmacological properties of *C. asiatica*. Antibacterial, antifungal, antiulcer, and antidiabetic activity and anti-inflammatory activity are some of them [81].

### 3.13. Curcuma longa

**Figure 11:**
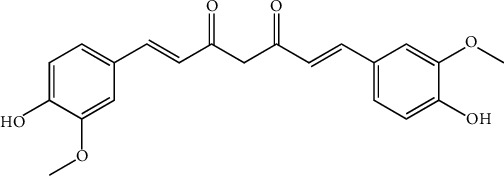
Structure of curcumin as the major constituent in *C. longa.*

*Curcuma longa* in which rhizome is commonly named as turmeric is an ancient coloring agent with various medicinal properties which belong to the family Zingiberaceae. The major phytochemicals found in *C. longa* is curcumin (Figure 11) with the chemical formula “1,7-bis (4-hydroxy-3-methoxyphenyl-1,6-heptadiene-3,5-dione).” The content of curcumin in *C. longa* varies with geographical features [87].

Curcumin as the major constituent in *C. longa* has a wide range of antiviral activity against many viral diseases. The bioconjugates of curcumin such as di-*O*-pamitoyl curcumin, di-*O*-bis-(*γ*, *γ*) folyl curcumin, di-*O*-tryptophanylphenylalanine curcumin, di-*O*-decanoyl curcumin, and C^4^-ethyl-*O*-*γ*-folyl curcumin have antiviral properties against parainfluenza virus type 3 (PIV-3), vesicular stomatitis virus (VSV), feline infectious peritonitis virus (FIPV), herpes simplex virus (HSV), respiratory syncytial virus (RSV), and Flock House virus (FHV) [87]. MTT assay results had depicted antiviral properties against different viral pathogens in further studies.

Apart from that, curcumin showed anti-influenza activity against influenza viruses PR8, H1N1, and H6N1. The test results showed a more than 90% reduction in virus yield in cell culture using 30 *μ*M of curcumin [88]. Curcumin as well as its derivatives show antiviral activity against herpes simplex virus type-1 (HSV-1) [89].

Human papillomaviruses (HPVs) are highly risk viruses, and the expression of E6 and E7 viral oncoprotein has the main role in cervical carcinoma. Curcumin which is the main constituent of *C. longa* has the inhibitory activity against the expression of E6 and E7 genes of two types of HPVs which are highly oncogenic human papillomaviruses [90]. According to the investigation of the antiviral function of curcumin on Neuro2 cell line infected with the Japanese encephalitis virus, the inhibition of the ubiquitin-proteasome system has resulted while reducing the occurrence of infectious particles [91]. Apart from that, curcumin exhibits antibacterial activity, synergistic antimicrobial activity, antibiofilm activity, and antifungal activity.

### 3.14. Woodfordia fruticosa

*Woodfordia fruticos* is commonly known as fire flame bush and Shiranjitea, and it belongs to the family *Lythraceae*. This plant can be seen most prevalently in Sri Lanka, India, Indonesia, Malaysia, China, and Japan. Various parts of these plants like stem, leaves, and flowers had been used from ancient times as medicine. *W. fruticosa* flower has been used for treating diarrhea, internal hemorrhages, dysentery, leucorrhoea, and menorrhagia [92].

The major constituent in flowers is tannins. Chrysophenol-8-O-*β*-D-glucopyranoside, *β*-sitosterol, cyanidin-3.5-diglucoside, and octacosanol also have been extracted from flowers. Leaves contain ellagic acid, myricetin-3-galactoside, pelargonidin-3,5-diglucoside, and polystachoside. The plant also contains trimeric and tetrametric hydrolyzable tannins [69, 93].

*W. fruticos* has many pharmacological properties such as antiulcer properties [94], immunomodulatory activity [95], hepatoprotective activity [96], antitumor activity [97], and wound healing activity [98]. During viral infection, reactive oxygen species are produced by the action of viruses which causes damages to the infected cells. Antioxidant which has the ability to reduce the reactive oxygen species exhibits some antiviral properties. Extracts of *W. fruticosa* flowers were found to have excellent antioxidants that trigger the antiviral activity [99].

At the same time, *W. fruticosa* has antiviral activity against enterovirus. Aqueous and methanolic extracts of *W. fruticosa* flowers and leaves inhibited avian myeloblastosis virus reverse transcriptase. Even at the extract concentration which shows 90% inhibition, there had been no cytotoxicity observed. Gallic acid present in flower extract exhibits antiherpes simplex type-1 virus and antihuman immunodeficiency virus activity [100].

### 3.15. Phyllanthus emblica

*Phyllanthus emblica* is a member of the *Euphorbiaceae* family, and it has widely distributed in most tropical and countries. The fruit of the *P. emblica* is one of the most powerful and widely used herbal medicines in the Ayurvedic and Unani medicinal systems [101]. Ascorbic acid (vitamin C) is the most abundant component of *P. emblica* fruit. Also, other phytochemicals extracted from this plant are included fixed oils, phosphates, essential oils, tannins, minerals, vitamins, amino acids, and fatty acids. The fatty acids identified by *P. emblica* are linolenic, linoleic, oleic, stearic, palmitic, and myristic acids. D-glucose, D-fructose, D-myositol, D-galacturonic acid, D-arabinose, D-rhamnosyl, D-xylosyI, D-glucosyI, D-mannosyl, and D-galactosyI residue are sugars. The major tannins identified in the plant are emblicanin A, emblicanin B, pedunculagin, and punicgluconin [102].

**Figure 12:**
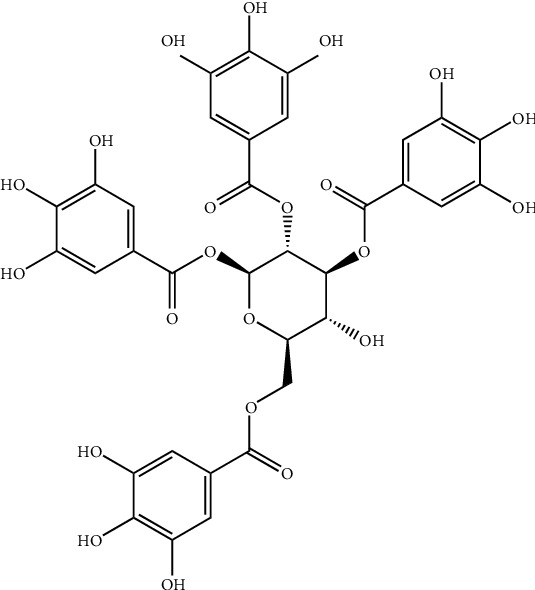
Structure of 1,2,4,6-tetra-O-galloyl-*β*-D-glucose, a polyphenolic compound isolated from *Phyllanthus emblica.*

A polyphenol compound isolated from *P. emblica*, named 1,2,4,6-tetra-O-galloyl-*β*-D-glucose (Figure 12), has been documented in vitro for antiviral activity against herpes simplex virus. It shows that the inhibition of herpes simplex virus type-1 (HSV-1) and HSV-2 is in different magnitudes. Further studies suggest this compound inhibited HSV-1 viral attachment in the early stage by blocking viral attachment and penetration. Additionally, due to the presence of 1,2,4,6-tetra-O-galloyl-*β*-D-glucose, viral protein synthesis processes also have been reduced [103].

Phyllaemblicin B and phyllaemblicin C and phyllaemblic acid methyl ester extracted from the roots of the *P. emblica* exhibited antiviral activity against coxsackievirus B3 (CVB3) in an *in vitro* cytopathic inhibitory assay. Half maximum inhibitory concentrations (IC50) have reported as 7.8 *μ*g/mL, 11.0 *μ*g/mL, and 21.8 *μ*g/mL for phyllaemblicin B, and phyllaemblicin C and phyllaemblic acid methyl ester, respectively, and ribavirin considered as control and its IC50 value reported as 20.3 *μ*g/mL explaining phyllaemblicin B, and phyllaemblicin C and phyllaemblic acid methyl ester are potential candidates for natural anticoxsackievirus B3 agents [104].

In the meantime, another study found that some of the highly oxygenated bisabolane sesquiterpenoid glycoside phyllaemblicins isolated from the *P. emblica* have possible antihepatitis B virus (HBV) activities [105]. In the roots of many *Phyllanthus* spp., bisabolane sesquiterpenoid glycosides which are highly oxygenated are present. In addition, the key components of the root extract of Phyllanthus emblica, phyllaemblicin B, and glochicoccinoside D showed potential antiviral activity against influenza A virus strain H3N2 and hand, foot, and mouth virus EV71 (H3N2 and EV71). Furthermore, the identified glochicoccinoside D exhibited potent antiviral efficacy against hand, foot, and mouth virus EV71 and influenza A virus strain H3N2 with IC_50_ values; 2.6 ± 0.7 mg/mL and 4.5 ± 0.6, respectively.

### 3.16. Terminalia chebula

*Terminalia chebula* is a native plant in India. It belongs to the family *Combretaceae*. It is a very common medicinal plant in Unani, Ayurveda, and homeopathy [106]. Rural folks used this plant to treat asthma, heartburn, vomiting, dysentery, sore throat, and ulcers. The main phytochemical constituents in *T. chebula* are tannins and polyphenols like ellagic acid, anthraquinones, chebulinic acid, corilagin, galloylglucose, and triterpenes [107].

*T. chebula* has antiviral properties. It has the ability to fight against the influenza A virus to protect the upper respiratory cells and also helps to prevent pulmonary infections [108]. In different studies regarding *T. chebula* constituents had demonstrated its ability to heal the herpes simplex virus [109]. In the literature, it has mentioned the ability of *T. chebula* to inhibit the activity of reverse transcriptase and protease of human immunodeficiency virus-1 [110]. Apart from that, a group of investigators looked at the antiviral activity of *T. chebula* on human cytomegalovirus (CMV) and plant extract of *T.chebula* was involved in the prevention of CMV replication in immune-compromised mice and determined that it could be useful to inhibit CMV infections in immune-compromised patients as well [111].

Tannins can be extracted from *T. chebula* and it has a pronounced effect in plant pathogenic potato virus [112]. And also according to the results of recent research, constituents such as chebumeinin A, chebumeinin B, and some other hydrolyzable tannins in the dried fruit of *T.chebula* have the possibility to react against hepatitis C virus [113].

### 3.17. Tamarindus indica

Tamarind or *Tamarindus indica* belonging to *Fabaceae*, a subfamily of *Caesalpinioideae*, is an important food in the tropics. It is a multipurpose tree, almost every part of which has at least some kind of nutritional or medicinal function. Tamarind is native to tropical Africa but has been spread to most of the world's regions [114].

Tamarind leaves are a fair source of vitamin C and carotene; the mineral content is high, especially P, K, Ca, and Mg. The antioxidant, anti-inflammatory, antimicrobial, and antifungal activity has been reported from many plant sections, and Tamarind fruit pulp has a sweet acidic taste due to a combination of high tartaric acid content and reducing sugar content [115]. Phenol-rich food and drinks including red wine, grapes seeds, green tea, and Tamarind have a hypolipidemic, antiatherosclerotic, antioxidant, anti-inflammatory, and immunomodulatory impact. In particular, the rich content of polyphenols in seed and fruit has a beneficial impact on neutrophils. *T. indica* fruit extract has been shown to inhibit watermelon mosaic virus, cowpea mosaic virus, and tobacco mosaic virus as an effective antiviral capability on plant-infected viruses [116].

Inhibition of virus replication and/or virucidal function of *T. indica*, stem bark, crude ethanol extract, and column chromatographic fractions tested for velogenic Newcastle disease virus (NDV) and showed positive activity, and minimum inhibitory concentrations of bark extract for virus inactivation was 0.24 mg/mL [117].

### 3.18. Terminalia arjuna

*Terminalia arjuna* initially was referred to as “Hirdya” a drug that strengthens the heart, but further research and clinical evidence indicate that it also possesses strong anticancer and antiviral activity [118].

Significant chemical components present in the plant triterpenoids are primarily responsible for cardioactive properties. Tannins are responsible for anticancer and antiherpes simplex virus (HSV) and polyphenols, including flavonols and flavonols. Phenylpropanoids are also useful for cancer treatment [119].

**Figure 13:**
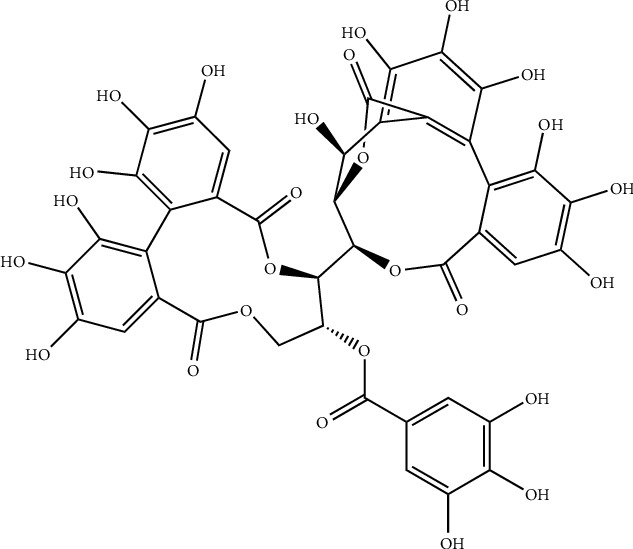
Structure of casuarinin, a hydrolyzable tannin isolated from the *Terminalia arjuna* bark.

Casuarinin (Figure 13), a hydrolyzable tannin isolated from the *T. arjuna* bark has antiviral activity on herpes simplex type 2 viruses (HSV-2) in vitro. IC50 value of casuarinin in sodium 30-[1-(phenylamino-carbonyl)-3,4-tetrazolium]-bis (4-methoxy-6 nitro) benzene sulphonic acid (XTT) and plate reduction assays were found to be 3.6 ± 0.9 and 1.5 ± 1.0 0 microgram. Casuarinin has also been shown to prevent HSV-2 from being bound to cells. It also exhibited active inhibition of viral penetration [120].

In the meantime, another research group performed a critical antiviral experiment using aqueous leaves extract *T. arjuna* to investigate its antigen inhibitory capability (Infectious Bursal Disease, IBD) of specific T cell populations evaluating the lymphocyte proliferation and CD14- monocyte surface markers in PBMCs (human peripheral blood mononuclear cells). The findings have exhibited that the aqueous leaf extract of *T. arjuna* showed a significant drop down in CD14 and IBD monocyte surface markers in PBMCs at higher doses [121].

### 3.19. Azadirachta indica

*Azadirachta indica* (neem) is a tree belonging to the *Meliaceae* family. It has been originated in southern and southeastern Asia. *A. indica* widespread in tropical and subtropical areas of Asia, Africa, America, and Australia. It is an evergreen, deciduous, fast-growing plant which can reach a height of 25 meters [122].

**Figure 14:**
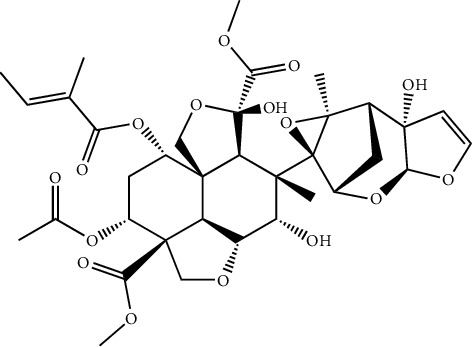
Structure of azadirachtin contains in the seed of the *Azadirachta indica* which recognize as major constituent for anti-intertidal properties.

Neem has been extensively used in indigenous medicinal systems such as Ayurveda by Indians for over 2000 years. It is traditionally used for the healing of various diseases [123]. Traditional use of *A.indica* as an antiviral efficacy to recommend for the treatment of bovine and avian poxvirus infected animals by applying leaf paste directly to infected animal skin [124]. Azadirachtin (Figure 14) contains the seed of the *Azadirachta indica* which recognize as a major constituent.

Badam et al. et al. reported that the methanolic extract of *A. indica* leaves inhibited the plaque formation of many serotypes of coxsackievirus B. In addition to flavonoids, triterpenoids, and their glycosides, the presence of a battery of compounds in the extract has antiviral activity against the coxsackie B virus community in vitro [125]. Neem oil demonstrated strong antiviral activity by preventing the replication of poliovirus [126]. In vitro, the antiviral activity of the aqueous neem leaf extract evaluated clone cells of larvae of Aedes albopictus using virus inhibition assay showed dose-dependent inhibition of dengue 2 viruses [127]. Mahmood et al. stated that the neem bark extract showed significant inhibition for Newcastle disease virus (NDV) [128]. An in silico docking study conducted by Ashfaq et al. revealed that phytochemicals present in *A. indica* leave having antiviral activity against HCV NS3 protease where 3-deacetyl-3-cinnamoyl-azadirachtin possesses appropriate binding properties with hepatitis C virus NS3/4A protease and it can be concluded that deacetyl-3-cinnamoyl-azadirachtin serves as a potential inhibitor against the studied protease [129].

### 3.20. Ficus religiosa

*Ficus religiosa* is a member of the family Moraceae [130]. It is considered the most sacred tree of South Asia, and different parts of the plant have been extensively used in traditional medicinal systems such as Ayurveda and Unani for various disorders [131]. *F. religiosa* is known by more than 150 names, and it is native to the sub-Himalayan tract, central India, and Bengal. It has been extensively distributed across the world through cultivation [132, 133]. Phytochemical studies carried out on *F.religiosa* revealed phytosterols, furanocoumarins, amino acids, phenolic components, aliphatic alcohols, hydrocarbons, volatile components, and some other classes of secondary metabolites isolated from the different parts of the plant. Phenolic components (flavonoids and tannins) and amino acids are found in almost all parts of the plant [132, 134].

*F. religiosa* extracts have been used in indigenous medicine to treat sexually transmitted diseases (STDs) such as gonorrhea and genital ulcers. Water and chloroform extracts of *F. religiosa* bark have been showing efficacy against herpes simplex virus type 2 (HSV-2) and, most notably, have demonstrated antiviral activity against acyclovir-resistant strain HSV-2. In addition, the water extract of *F. religiosa* bark has the ability to direct inactivating virus activity. Overall, the extract of chloroform interferes with viral attachment and viral entry as well as restricts the generation of viral progeny [131]. Cagno et al. reported that *F. religiosa* methanolic bark extract yielded good antiviral action against human rhinovirus whereas this extract inhibited the late steps of the viral replicative cycle. The aqueous bark extract was the most effective as an antirespiratory syncytial virus. Both partial viral inactivation and virus attachment interference were found to be responsible for the anti-RSV activity. The replication of both tested viruses was inhibited [130].

Table 2 summarized the importance of secondary metabolites extracted from the selected medicinal herbs and antiviral mechanisms revealed by the different research studies. Those studies would be the initial steps for discovering of effective drugs against viral diseases.

## 4. Conclusions

Most significant medicinal plants which are frequently used as antiviral treatments in Ayurveda medicine have been subjected to recent research studies, and specifically, bioactive compounds have been isolated and identified as antiviral phytocompounds against different human infected viral species. Gingerols extracted from *Zingiber officinale* as the major phytocompound would be the most significant phytocompound inhibiting HRSV activities, computational investigation has discovered that Taepeenin J can be obtained from *Caesalpinia bonducella* as a promising candidate for receptor inhibition process to mitigate cytokine storms due to the infection of the SARS-CoV-19 virus, ajoene and allicin extracted from the *Allium sativum* have exhibited for significant anti-HIV and antiherpes simplex virus capabilities, respectively, glycyrrhizin from the *Glycyrrhiza glabra* is more effective in the regulation of viral replication and can be used as a prophylactic measure, and it was also used to prevent the replication of the severe acute respiratory syndrome (SARS) coronavirus. The key pigment of cotton seed was gossypol, a phenolic compound that has antiviral properties against enveloped viruses, including HIV-1, HSV-2, influenza, and parainfluenza. A polyphenol compound isolated from *Phyllanthus emblica*, named 1,2,4,6-tetra-O-galloyl-*β*-D-glucose, has been documented in vitro for antiviral activity against herpes simplex virus, casuarinin, a hydrolyzable tannin isolated from the *Terminalia arjuna* bark, has antiviral activity on herpes simplex type 2 viruses (HSV-2), and sesquiterpene cumarines isolated from the oligo-gum resin of *Ferula assafoetida* showed antiviral properties against rhinovirus (HSV) and influenza A H1N1 virus. Additionally, water and other solvent extracts of the selected herbs have exhibited critical antiviral activities against different disease-forming viral species. Hence, more studies need to be focused on the bioactive constitutes of the medicinal herbs for developing as antiviral drugs.

## Figures and Tables

**Table 1:** Preclinical investigations of viral inhibition assays of organosulfur compounds which isolate from *A. sativum.*

Organosulfur compound	Detection method	Virus species	Antiviral mechanism	Reference
Allicin	Direct preinfection incubation and plaque reduction assay	Herpes simplex virus-1 Herpes simplex virus-2 Parainfluenza virus-3 Vaccinia virus Vesicular stomatitis virus Human rhinovirus type 2	Disruption of viral envelop and cell membrane	[32]

Alliin	Anti-inflammatory assay	Dengue virus	Inhibition of inflammation via reduction of oxidative stress	[36]

Ajoene	Anti-HIV activity	Human immunodeficiency virus-1	Prevention of induced destructions of CD+ cells	[32]
	HIV-infected platelet aggregation and fusion assays	Human immunodeficiency virus	Inhibition of adhesive bonds and fusion of leukocytes	[37]
	HIV induced cellular toxicity assay	Human immunodeficiency virus-1	Inhibition of viral reverse transcriptase and cell attachment	[38]

Allyl methyl thiosulfinate	Direct preinfection and plaque reduction assay	Herpes simplex virus-1 Herpes simplex virus-2 Parainfluenza virus-3 Vaccinia virus Vesicular stomatitis virus Human rhinovirus type 2	Disruption of viral envelop and cell membrane	[32]

Diallyl trisulfide	Plaque reduction assay	Human cytomegalovirus; IAV-H1N1	Inhibit viral DNA synthesis through inhibition of HCMV immediate early antigen expression	[39]

Diallyl sulfide and Diallyl disulfide	Oxidative stress and anti-inflammatory assay	Dengue virus	Reduction of oxidative stress	[36]

**Table 2:** Summary (phytocompounds and antiviral mechanisms).

Phytocompound	Medicinal herb	Target viral species	Mechanism
(1) Gingerols	*Zingiber officinale*	HRS virus	N/I
(2) Taepeenin j	*Caesalpinia bonducella*	SARS-COV-19 virus	Mitigate cytokine storms by preventing the receptor binding process
(3) Allicin	*Allium sativum*	Herpes simplex virus-1 Herpes simplex virus-2 Parainfluenza virus-3 Vaccinia virus Vesicular stomatitis virus	Disruption of viral envelop and cell membrane
(4) Alliin and diallyl sulfide	*Allium sativum*	Dengue virus	Reduction of oxidative stress
(5) Ajoene	*Allium sativum*	Human immunodeficiency virus-1	Inhibition of viral reverse transcriptase and cell attachment
(6) Allyl methyl thiosulfinate	*Allium sativum*	Herpes simplex virus-1 Herpes simplex virus-2 Parainfluenza virus-3 Vaccinia virus Vesicular stomatitis virus Human rhinovirus type 2	Disruption of viral envelop and cell membrane
(7) Diallyl trisulfide	*Allium sativum*	Human cytomegalovirus; IAV-H1N1	Inhibit viral DNA synthesis
(8) Glycyrrhizin	*Glycyrrhiza glabra*	Herpes simplex virus (HSV-1 and HSV-2) and severe acute respiratory syndrome (SARS) coronavirus	Inhibit the plaque formation of herpes simplex virus (HSV-1 and HSV-2) Prevent the replication of the severe acute respiratory syndrome (SARS) coronavirus
(9) Microlobidene, Farnesiferol C, Farnesiferol B, Kellerin	*Ferula assafoetida*	Rhinovirus (HRV-2)	Prevention of rhinovirus adsorption (HRV-2)
(10) 8′-acetoxy-5′S-hydroxyumbelliprenin, methyl galbanate, galbanic acid, conferol	*Ferula assafoetida*	Influenza A H1N1 virus	N/I
(11) Gossypol	*Gossypium herbaceum*	(HIV-l) human immunodeficiency	Prevented the replication
(12) Repandusinic acid	*Phyllanthus niruri*	(HIV-l) human immunodeficiency	Inhibitory effect on HIV-1 reverse transcriptase
(13) Withaferin A	*Withania somnifera*	Herpes simplex virus	Inhibits herpes simplex virus's DNA polymerases
(14) Curcumin	*Curcuma longa*	Influenza viruses PR8, H1N1, and H6N1	N/I
(15) 1,2,4,6-Tetra-O-galloyl-*β*-D-glucose	*Phyllanthus emblica*	Herpes simplex virus HSV-1	Blocking viral attachment and penetration
(16) Casuarinin	*Terminalia arjuna*	Herpes simplex type 2 viruses (HSV-2)	Inhibition of viral penetration

N/I: not identified.
